# Hydrogen sulfide as a therapeutic candidate in diabetic retinopathy: a new perspective from gas medicine

**DOI:** 10.4103/mgr.MEDGASRES-D-25-00186

**Published:** 2026-04-11

**Authors:** Mengting Xue, Wufei Dai, Songtao Yuan, Claudia Günther

**Affiliations:** Department of Medicine 1, Universitätsklinikum Erlangen and Friedrich-Alexander-Universität Erlangen-Nürnberg, Erlangen, Germany; Deutsches Zentrum Immuntherapie (DZI), Friedrich-Alexander-Universität Erlangen-Nürnberg (FAU), Erlangen, Germany; Department of Ophthalmology, Jiangyin Hospital of Traditional Chinese Medicine, Wuxi, Jiangsu Province, China; Department of Ophthalmology, The First Affiliated Hospital of Nanjing Medical University, Nanjing, Jiangsu Province, China

Diabetic retinopathy (DR) remains a leading cause of blindness, with limited treatment options targeting its underlying pathophysiology. This perspective highlights the promising yet underexplored role of hydrogen sulfide (H_2_S) as a gasotransmitter in mitigating retinal vascular dysfunction, oxidative stress, and inflammation in DR. By integrating recent preclinical evidence, we propose a novel framework for H_2_S-based therapies, bridging gaps between gas medicine and ophthalmology. We also discuss conventional approaches and encourage further research into this innovative therapeutic avenue.

**Figure 1:**
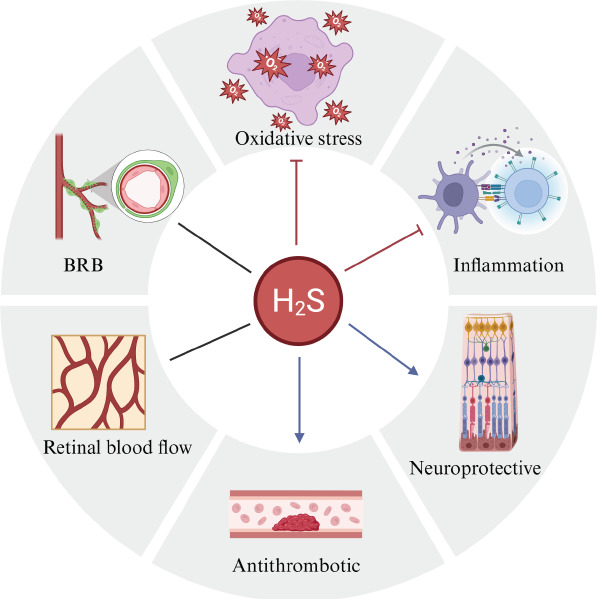
Potential therapeutic role of H_2_S in diabetic retinopathy (DR). Schematic representation of the multifaceted effects of H_2_S on DR. H_2_S exhibits antioxidant and anti-inflammatory effects. Furthermore, H_2_S exerts neuroprotective effects by preventing apoptosis and antithrombotic effects by reducing platelet aggregation, cell adhesion, and coagulation. Additionally, H_2_S shows the ability to stabilize the BRB by regulating the vascular permeability and pro-angiogenic factors, which depend on its concentration. Last but not least, H_2_S regulates the retinal blood flow by demonstrating dual vasoconstrictive and vasodilatory effects. Created with BioRender.com. BRB: Blood–retinal barrier; H_2_S: hydrogen sulfide.

DR is a major complication of diabetes mellitus and a leading cause of vision loss in the working-age population. Studies have shown that oxidative stress and inflammation are closely associated with DR.^1^ Current treatment strategies for DR primarily focus on managing microvascular complications, including intravitreal pharmacotherapy, laser photocoagulation, and vitrectomy. However, in clinical practice, the use of anti-vascular endothelial growth factor (VEGF) drugs is limited due to the need for frequent injections, financial burden, and poor patient compliance, with most patients failing to achieve clinically significant visual improvement. Meanwhile, conventional laser therapy can only stabilize vision. Therefore, there is an urgent need to develop novel therapeutic approaches. Growing evidence suggests that H_2_S exhibits antioxidant, anti-inflammatory, and vasoprotective properties,^2^ indicating its potential therapeutic role in DR (**Figure 1**).

**Source of H_2_S in the eye:** H_2_S is a gaseous signaling molecule in mammals, synthesized by pyridoxal-5’-phosphate-dependent enzymes including cystathionine β-synthase, cystathionine γ-lyase (CSE), as well as 3-mercaptopyruvate sulfurtransferase (3MST) and cysteine aminotransferase (CAT). These enzymes are differentially distributed in ocular tissues, with CSE predominantly localized in the retinas of amphibians and mammals, where its activity can be reliably measured. The 3MST/CAT pathway serves as the predominant route for H_2_S generation in mammalian retinas, given the co-localization of both 3MST and CAT within retinal neurons.

Similar to nitric oxide (NO) and carbon monoxide, endogenously produced H_2_S is now recognized as a crucial gasotransmitter that modulates protein structure and function through transient covalent modifications (such as S-sulfhydration, polysulfide formation, mental center modification, cross-talk with S-nitrosothiols and disulfide bond reduction or rearrangement). This gaseous mediator readily diffuses across cell membranes without requiring specific degradation or reuptake mechanisms. While high concentrations of H_2_S exhibit neurotoxicity and tissue damage in humans, physiological levels demonstrate significant vasoprotective potential.

**Therapeutic effects of H_2_S on diabetic retinopathy:** H_2_S exhibits direct or indirect antioxidant and anti-inflammatory effects. Under hyperglycemic conditions, glucose undergoes non-enzymatic condensation with the amino termini of proteins, leading to the accumulation of advanced glycation end products. This process disrupts the inner blood–retinal barrier (BRB), triggers oxidative stress responses and inflammation, and ultimately accelerates the progression of DR. H_2_S exerts protective effects by enhancing galactose metabolism, reducing advanced glycation end product formation in neuronal cells, and attenuating excessive oxidative stress. These mechanisms collectively mitigate the pathogenic role of advanced glycation end products in DR development.

Furthermore, in animal models of DR, H_2_S directly suppresses intracellular reactive oxygen species accumulation and associated excessive lipid peroxidation.^3^ One of the anti-inflammatory properties of H_2_S is its ability to scavenge pro-inflammatory oxidants such as ONOO−, HOCl, superoxide, and hydrogen peroxide. It also modulates inflammatory cytokines by inhibiting nuclear factor kappa-B activation.^4^

H_2_S exerts neuroprotective effects on neurons. In the pathological progression of DR, neuronal damage often occurs early and accumulates into visible retinal vasculopathy. In spinal cord ischemia–reperfusion injury, the slow-releasing H_2_S donor GYY4137 can ameliorate spinal cord neuron loss and exert a neuroprotective effect via the inhibition of apoptosis and overactivated microglia-mediated neuroinflammation.^5^

H_2_S has multiple effects on retinal vasculature, including stabilizing the BRB, exerting antithrombotic effects, and participating in retinal blood flow regulation. BRB dysfunction is a major contributor to retinal vascular lesions in DR. Exogenous H_2_S treatment in streptozotocin-induced diabetic rats reduces retinal BRB permeability, decreases acellular capillaries, lowers vitreous VEGF levels, down-regulates expressions of VEGF receptor 2 and hypoxia-inducible factor 1α, and increases occludin expression.^6^ Beyond inflammation and apoptosis, platelet adhesion also contributes to diabetes-induced retinal endothelial dysfunction. H_2_S plays a potential role in inhibiting fibrinogen binding and platelet activation, while also mitigating oxidative stress and promoting macrophage polarization toward the anti-inflammatory M2 phenotype, thereby exerting antithrombotic effects.^7^ Additionally, it demonstrates dual vasoconstrictive and vasodilatory effects through pathways such as adenylate cyclase/cyclic adenosine monophosphate and endothelial nitric oxide synthase-activated NO/cyclic guanosine monophosphate signaling, thereby protecting blood vessels, regulating blood pressure, and mitigating vascular inflammation.^8^

**Perspective developments of H_2_S in diabetic retinopathy:** Currently, various gasotransmitter-based strategies are being investigated as potential therapeutic approaches for vascular dysfunction. However, these strategies have not yet been explored in the context of DR-related vascular disorders. Given that H_2_S exhibits anti-inflammatory and antioxidant effects, inhibits apoptosis, and restores retinal microcirculatory homeostasis, it represents a promising therapeutic target for DR.

Nevertheless, due to the complexity of BRB and the unique anatomical structure of the eye, the administration route of H_2_S must be carefully considered. In streptozotocin-induced diabetic rats, treatment with the H_2_S donor sodium hydrosulfide improved vasodilation and NO bioavailability, suggesting that H_2_S may serve as a potential therapeutic agent for DR through crosstalk with NO.^9^

However, the precise therapeutic and pathological concentrations of H_2_S remain elusive. In recent years, novel H_2_S-releasing drugs such as ATB-346 and ATB-352 have demonstrated efficacy in treating gastrointestinal disorders and hold broad potential for application in various ocular diseases. Wang et al.^10^ demonstrated that diallyl trisulfide-loaded mesoporous silica nanoparticles could release H_2_S, promoting endothelial cell proliferation and migration while exerting cytoprotective effects, reducing inflammatory cytokine production, and suppressing adhesion molecule expression. Additionally, H_2_S-sustained-release biomaterials such as poly(3,4-ethylenedioxythiopene)-assembled polydopamine-mediated silk microfiber network have been shown to enable the hydrogel to reverse the inflammatory microenvironment, creating a pro-regenerative microenvironment in a hyperglycemic inflammatory microenvironment.^11^

When considering H_2_S as a therapeutic target, reliable methods are required to determine its levels in biological fluids and tissues. However, quantification of H_2_S remains challenging due to its volatile nature. As an alternative, stable end products such as sulfate or thiosulfate can be measured in serum or urine.

The methylene blue assay represents the most commonly employed technique. This method relies on the oxidative coupling of H_2_S with two molecules of N, N-dimethyl-p-phenylenediamine to form methylene blue dye, which can be detected spectrophotometrically. Nevertheless, this technique demonstrates significant pH dependence and exhibits limited sensitivity and reliability.

A more sensitive approach utilizes monobromobimane, where two monobromobimane molecules form stable sulfide-dibimane in the presence of H_2_S. The resulting sulfide-dibimane can be separated by reverse-phase chromatography and detected using a fluorescence detector. Currently, fluorescent probes and sulfide-selective electrodes are widely used, though their sensitivity and reliability vary considerably.^12^

To date, the measurement of H_2_S remains technically difficult with inconsistent reliability, thereby complicating research on its biological functions and potential as a therapeutic target in various diseases, including diabetes-associated vascular disorders. Therefore, accurate measurement of H_2_S concentrations remains a critical direction for future research.

**Future directions:** In future research on H_2_S, the focus lies on the rate, location, and duration of H_2_S release from its donors. Therefore, further exploration is needed to monitor and regulate both endogenous and exogenous sources of H_2_S concentration. Additionally, more efficient H_2_S-releasing compounds must be developed, and a deeper understanding of their chemical properties and mechanisms should be pursued. Here are several specific future directions for H_2_S-based therapy in DR.

The development of H_2_S-based therapies for DR requires addressing several key challenges while exploring novel therapeutic avenues. Current research directions focus on optimizing drug delivery systems to overcome ocular bioavailability limitations. Nanoparticle-based carriers, including lipid and polymeric formulations, show promise for targeted H_2_S donor delivery, while stimuli-responsive intravitreal implants may enable sustained release modulated by the local DR microenvironment (e.g., hyperglycemia or oxidative stress). These advanced delivery systems must be designed to maintain therapeutic H_2_S concentrations while minimizing potential toxicity.

A particularly promising approach involves the development of combination therapies that integrate H_2_S donors with existing DR treatments. The synergistic potential of H_2_S with anti-VEGF agents may address the limitations of current monotherapies by simultaneously targeting multiple pathological pathways, including angiogenesis, inflammation, and oxidative stress. Similarly, combining H_2_S with neuroprotective agents could provide comprehensive retinal protection by preserving both vascular and neuronal components. Future studies should systematically evaluate these combination strategies in relevant preclinical models to identify optimal dosing regimens and potential additive or synergistic effects.

Significant opportunities exist in modulating endogenous H_2_S production through enzyme-targeted approaches. The differential expression of H_2_S-synthesizing enzymes (CSE, cystathionine β-synthase, and 3MST/CAT) in retinal cell types suggests that selective activation of specific enzymatic pathways could achieve cell-type-specific therapeutic effects. Small molecule activators of 3MST/CAT or gene therapy approaches using adeno-associated virus vectors to upregulate CSE expression represent potential strategies to enhance local H_2_S production. Concurrently, the development of reliable biomarkers for H_2_S activity in ocular tissues remains crucial for monitoring therapeutic efficacy and establishing dose-response relationships in clinical settings.

Emerging technologies, including smart biomaterials with microenvironment-responsive H_2_S release properties and AI-driven drug discovery platforms, may accelerate the development of next-generation H_2_S donors. Light-activated compounds and glucose-sensitive H_2_S donors offer the potential for precise spatiotemporal control of H_2_S delivery. Furthermore, integrating multi-omics approaches (proteomics, metabolomics, and single-cell transcriptomics) could provide comprehensive insights into H_2_S-mediated signaling networks in DR. As research progresses, clinical translation will require careful evaluation of safety profiles and therapeutic windows, as well as standardization of H_2_S detection methodologies to facilitate comparative analysis across studies.

These developments position H_2_S-based therapy as a promising multi-target approach for DR, potentially benefiting patients with suboptimal responses to current treatments. Future research should prioritize the translation of preclinical findings into clinical trials while addressing the unique pharmacokinetic and pharmacodynamic challenges of ocular H_2_S delivery. The integration of H_2_S therapeutics into the DR treatment paradigm may ultimately provide a much-needed complementary strategy to address the multifactorial nature of this sight-threatening complication.

**Conclusion:** H_2_S exhibits multifaceted therapeutic effects in DR, including antioxidant, anti-inflammatory, cytoprotective, and vasoprotective properties, making it a promising novel therapeutic target, particularly for patients who are insensitive to anti-VEGF therapy. The development of advanced controlled-release H_2_S technologies, coupled with precise H_2_S concentration monitoring techniques, opens new prospects for biomedical applications, particularly in the fabrication of slow-releasing H_2_S biomaterials. These innovations present a significant opportunity to enhance treatment efficacy and improve clinical outcomes in DR patients with suboptimal therapeutic responses.
